# Factors associated with the utilization of institutional delivery services in Bangladesh

**DOI:** 10.1371/journal.pone.0171573

**Published:** 2017-02-13

**Authors:** Sanni Yaya, Ghose Bishwajit, Michael Ekholuenetale

**Affiliations:** 1 School of International Development and Global Studies, Faculty of Social Sciences, University of Ottawa, Ottawa, Ontario, Canada; 2 School of Medicine and Health Management, Tongji medical College, Huazhong University of Science and Technology, Wuhan, Hubei, China; 3 Women’s Health and Action Research Centre, Benin City, Nigeria; Centre for Injury Prevention and Research, BANGLADESH

## Abstract

**Introduction:**

Bangladesh has made remarkable progress towards reducing its maternal mortality rate (MMR) over the last two decades and is one of the few countries on track to achieving the MMR-related Millennium Development Goals (MDG-5A). However, the provision of universal access to reproductive healthcare (MDG-5B) and the utilization of maternal healthcare services (MHS) such as institutional delivery, which are crucial to the reduction of maternal mortality, are far behind the internationally agreed-upon target. Effective policymaking to promote the utilization of MHS can be greatly facilitated by the identification of the factors that hinder service uptake. In this study, we therefore aim to measure the prevalence of institutional delivery services and explore the factors associated with their utilization in Bangladesh.

**Methods:**

Data for this study were extracted from the 2011 Bangladesh Demographic and Health Survey (BDHS, 2011); participants were 7,313 women between the ages of 15 and 49 years, selected from both urban and rural households. Data were analyzed using Chi-square analysis, and conditional logistic regression.

**Results:**

According to the findings, fewer than one in three women reported delivering at a health facility. The multivariable regression analysis showed that participants from rural areas were 46.9% less likely to have institutional deliveries compared to urban dwellers (OR = 0.531; p<0.001; 95%CI: 0.467–0.604), and participants aged between 30 and 49 years had a 23.6% higher prevalence of institutional delivery service utilization compared to those aged 15 to 29 years (OR = 1.236; p = 0.006; 95%CI: 1.062–1.437). Moreover, participants with higher educational attainment were about twice as likely to deliver at a standard health facility when compared to those without formal education (OR = 2.081; p<0.001; 95%CI: 1.650–2.624), and similarly, husbands with higher educational attainment exhibited an approximately 71% higher service utilization of institutional delivery facilities compared to those without formal education (OR = 1.709; p<0.001; 95%CI: 1.412–2.069). Wealth status was also a significant predictor of institutional delivery service use, with participants belonging to the highest economic stratum being more likely to receive skilled care compared to the lowest economic stratum (OR = 2.507; p<0.001; 95%CI: 2.118–2.968). In addition, results indicated that households of average economic class had a 27% higher level of institutional delivery service utilization compared to those of lower economic status (OR = 1.272; p = 0.011; 95%CI: 1.057–1.531). Furthermore, institutional health service use was 18% higher among participants who were aware of community clinical services compared to those who were hardly aware of these services (OR = 0.816; p = 0.012; 95%CI: 0.696–0.957). Lastly, the odds of utilizing delivery services was 1.553 times more likely for participants who use family planning compared to those who do not (p<0.001; 95%CI: 1.374–1.754), and 3.639 times more likely for those who receive antenatal care compared to those who do not (p<0.001; 95%CI: 3.074–4.308). These were found to be significant predictors of the choice of delivery services.

**Discussion:**

Our results suggest that efforts towards reducing national maternal mortality in Bangladesh could be aided by investments into education, poverty reduction and the strengthening of reproductive healthcare services through community clinics, with particular focus on rural areas.

## Introduction

The fifth Millennium Development Goal (MDG) adopted in 1990 was dedicated to the reduction of maternal mortality by three-quarters by 2015 [1]. Notably, immense progress has been made in this respect since then [2] however, the incidence of maternal mortality remains significant in developing countries [3]. Low- and middle-income countries (LMICs) altogether account for a staggering 99% of worldwide cases of maternal mortality, of which an estimated 80% are considered avoidable [4]. In addition to the high maternal mortality rate (MMR), another significant challenge for promoting maternal health in the LMICs is increasing the provision and utilization of essential maternal healthcare services (MHS).

About 98% of women in developed countries receive antenatal care (ANC) and up to 94% of births are attended by skilled health professionals [5]. In LMICs on the other hand, about half of all women are deprived of adequate ANC [6]. Utilization of MHS such as family planning, antenatal and prenatal care, and institutional delivery have proven to be significantly correlated with reductions in maternal mortality [7,8]. Yet, an overwhelming majority of women in developing countries lack access to these services and about half of them choose to give birth outside health institutional settings in unsafe and unhygienic conditions [9]. The availability of life saving equipment and hygienic conditions are vital to minimizing the risk of delivery complications and thus ensuring the health and wellbeing of the mother and her child [10]. Identifying and addressing the underlying factors that prevent MHS utilization is therefore paramount to combating maternal mortality and morbidity in resource poor settings and designing effective maternal health interventions.

A review of the existing literature on the subject reveals a growing research focus on examining the factors that affect maternal healthcare-seeking behaviour [11], with previous studies indicating that such care-seeking behaviour is influenced by various personal, sociocultural and environmental factors, such as individual perceptions of health, self-efficacy, motivation, social values and belief systems [12,13]. However, evidence-based research in this context is scarce in the case of Bangladesh. To this end, we attempt to identify factors associated with institutional delivery service use in Bangladesh by utilizing data from the 2011 demographic and health survey.

Historically, Bangladesh is characterized by high fertility and maternal mortality rates, (412/100000 pregnancies in 1976) [14] with a high burden of unsafe abortion-related complications [15]. About 71% of total births in Bangladesh occur at home [9] and merely one in five women (2007 estimate) is attended to by a skilled health care provider during delivery [16], which is a proportion far below the internationally set target of 85% by 2010 and 90% by 2015 [17]. A major barrier for meeting this target is an acute human resource crisis in the healthcare system in Bangladesh [18]. This shortage of health care professionals (HCPs) leads to a higher dependency on traditional or less qualified service providers, especially among remote and marginalized communities in rural areas. With an aim to tackle this issue, the Bangladeshi ministry of health launched a nationwide community clinic project entitled ‘Revitalization of Community Health Care Initiatives in Bangladesh (RCHCIB)’ in 2009, and made it a top priority project in the public health sector. Operating at the grassroots level, these community health centre initiatives provide first aid, nutrition and health education, family planning and primary care services. However, barriers to maternal healthcare access persist, especially in rural areas of the country, where there is a lower concentration of reproductive care facilities and healthcare professionals compared to urban areas. Compounded by poor health infrastructure and low resource availability, the country therefore faces extraordinary challenges to ensure universal access to reproductive health services and to meet the requirement set by MDG5 of ensuring the presence of a skilled birth attendant at every birth.

Though traditional birth attendants (TBAs) are cheaper and are more frequently available sources of delivery service for poorer households, the services they provide are not as effective or safe as those offered by skilled health care providers, since they are usually underequipped, lacking the appropriate technologies to handle critical birth-related complications (e.g. postpartum hemorrhage/PPH) [19]. In addition, even when skilled care is available, the likelihood of service uptake is low or inadequate due to unaffordability, poor maternal health literacy and the comparatively cheaper availability of traditional birth attendants (TBAs). In neighboring countries such as India, where MMR is also notably high, poverty and illiteracy are shown to be some of the most important predictors of MMR and of poor utilization of MHS [20]. However, for Bangladesh, knowledge regarding the factors associated with utilization of delivery services is very limited. Therefore, the present study aims to address this knowledge gap and explore the factors that are associated with the uptake of skilled delivery services in Bangladesh. The findings are also expected to assist in making programmatic efforts to promote skilled care delivery in the country.

## Methodology

### Data source, study area, and sampling procedure

Data for this study were extracted from the sixth round of the Bangladesh Demographic and Health survey (BDHS) collected from the DHS program. The DHS survey is nationally representative, cross-sectional in nature and was carried out in 2011 from July 8^th^ through December 27^th^. The Bangladesh survey was conducted by a local research institute (National Institute of Population Research and Training/NIPORT) as part of the International Demographic and Health Survey program known as MEASURE DHS, which is currently active in 90 countries and conducted under the auspices of the United States Agency for International Development (USAID) with the technical assistance of ICF International, based in the USA. The Demographic and Health Surveys (DHSs) are free, public datasets, though researchers must register with MEASURE DHS and submit a request before access to DHS data is granted. This data request system ensures that all users understand and agree to basic data usage ethics standards. The 2011 BDHS covered 17,141 households in total.

The survey employed a two-stage cluster sampling method covering the population residing in non-institutional dwelling units in Bangladesh. The country has seven administrative regions, which are subdivided into zilas (districts) and upazilas (townships). At the urban level, an upazila is divided into union parishads and mouzas (subdivision of union parishads), and at the rural level into wards and mohallas (subdivision of wards). The two-stage clustering of the population involved labeling the smallest administrative units as enumeration areas (EAs) or clusters, each consisting of households at mouza or mohalla level. Firstly, EAs were selected based on their size proportional to that of the units. In the second stage, households were selected systematically from each EA to ensure effective sampling. DHS provides no exact information on the spatial dimension of EAs. However, they consist of about one hundred to thirty thousand households varying from country to country.

### Variables selection and measurement

#### Dependent variable

The dependent variable was the place of delivery and was dichotomised in the following way: (1) Institutional (For deliveries occurring at a Government hospital, Special medical college, District hospital, Maternal and child welfare center/MCWC, Upazilla health complex, Health and family welfare center, Private hospital/clinic, Private medical college hospital); and (2) Non-Institutional (For deliveries occurring at respondents’ or relatives’ homes, or in other nonprofessional facilities)

Covariates (Socioeconomic and demographic) were categorized in the following way: Age: 15-29/30-49; Residency: Urban/Rural; Educational attainment of participant: Nil (no formal education), Primary (1–5 years of formal schooling), Secondary/Higher (>5 years of formal schooling); Employment status: Yes/No; Educational attainment of husband: Nil (no formal education), Primary (1–5 years of formal schooling), Secondary/ Higher (>5 years of formal schooling); Type of profession of husband**: Farming, Blue-collar and white-collar profession; Microcredit borrower (Yes/No) (based on membership with any of the following institutions):

1. Association for Social Advancement aka ASA
2. Bangladesh Rural Advancement Committee aka BRAC
3. Bangladesh Rural Development Board aka BRDB
4. Grameen Bank;

Household Wealth status: {Lowest (below average)}, {Middle (average)}, Highest {(above average}; Sex of household head: Male and Female; Has a say in household spending Yes/No; Has a say in own healthcare decision: Yes/No; Received Antenatal care: Yes/No. Aware of community clinic: Yes/No; involved in family planning: Yes/No; Heard about family planning on radio: Yes/No; Heard about family planning on TV: Yes/No; Heard about family planning in newspaper/magazine: Yes/No.

**Type of occupation was categorized in the following way: 1) Farming = Farmer, agricultural worker, angler, poultry farmer, cattle raising; Blue-collar jobs = carpenter, mason, driver, construction worker, rickshaw puller, brick breaking, road building; White-collar jobs = Manager, Businessman, physician, lawyer, accountant, teacher, government service holder.

#### Conditional logistic regression

Bangladesh has significant national disparities in fertility, with fertility rates varying widely between administrative divisions. Fertility was lowest in Khulna division (1.9 births per woman), followed by Rajshahi and Rangpur at 2.1 births per woman, and highest in Sylhet (3.1 births per woman) and Chittagong (2.8 births per women) (BDHS, 2011). Analysis involving matched designs is commonly used in situations where both the outcome probability and the factor of interest depend on a common set of variables, with matching variables being used so that the true relationship between the outcome and predictors is not confounded. Demographic variables are commonly used as matching variables. Within each stratum, samples of cases (institutional delivery = 1) and controls (non-institutional delivery = 0) are chosen. The number of cases and controls need not be constant across the strata, but the most common matched designs include one case and M controls per stratum and are thus referred to as 1:M matched studies. Conditional logistic regression works in nearly the same way as regular logistic regression. In this study, region is used as a matching variable, because this is known to be a confounder that influences both the covariates and the response.

A measure of the model fit for the conditional logistic regression model used in this study is the Negelkerke Pseudo R^2^. It is a measure intended to mimic the R-squared analysis. Although the interpretation is not the same as that of R^2^ analysis, it can be interpreted as an approximate variance in the outcome accounted for by the independent variables. This statistic, which is usually identical to the standard R^2^ when applied to a linear model, is generally either entropy-based or variance-based, where an entropy-based R^2^ statistic is also called pseudo-R^2^. The pseudo-R^2^ statistic is defined as the proportion of the variance of the latent variable that is explained by the predictor variables. This value tends to be smaller than R^2^ and values above 0.3 are considered highly satisfactory.

## Data analysis

The baseline socio-demographic characteristics of participants were tabled using percentages and Chi square (**χ**^**2**^**)** analysis was performed to check for factors that were associated with the choice of delivery location. Variables that were found to be significantly associated with delivery location from **χ**^**2**^ tests were entered into the regression model. Conditional logistic regression was used to adjust for confounders, and a *p*-value of < 0.05 was considered statistically significant for all associations. Data analysis was performed utilizing SPSS version 20 for Mac (SPSS Inc. Chicago. IL. USA).

## Ethical clearance

Before each interview, all participants gave informed consent to take part in the survey. The DHS Program maintains strict standards for ensuring data anonymity and protecting the privacy of all participants. ICF International ensures that the survey complies with the U.S. Department of Health and Human Services regulations for the protection of human subjects, whilst the host country ensures that the survey complies with local laws and norms. Further approval for this study was not required since the data is secondary and is available in the public domain. More details regarding DHS data and ethical standards are available at: http://goo.gl/ny8T6X.

## Results

### Baseline characteristics

Table 1. shows that three-fourths of the participants were aged between 15 and 29 and about one-third were of urban origin. Literacy rate was higher among women compared to husbands (81.8% vs 73.2%). Primary education completion rate was close to 30% among both women and husbands, however, women had higher secondary/higher education completion rates. About one in seven women reported working outside the home and a quarter were microcredit borrowers. The majority of the households were male-headed (89.6%), and most of them were engaged in blue-collar jobs (43.9%) rather than farming (26.3%) or white-collar jobs (29.8%). Two-fifths of the women were from poor households and another 40% were from rich ones. Women’s involvement in the decision-making process was quite low, as only about one in ten reported making health decisions for themselves and one in twenty reported having a say in household expenditure. About four-fifths of the participants reported being aware of community clinic services and two-thirds reported receiving antenatal care during pregnancy. A little over two-fifths of the women reported any family planning. Television (25.8%) was the most popular media for learning about family planning compared to radio (2.7%) and newspaper (3.6%).

**Table 1:** Basic Socioeconomic and demographic characteristics of the study population.

Variables	Frequency	Percent
**Age**		
15–29	5549	75.9
30–49	1764	24.1
**Residency**		
Urban	2326	31.8
Rural	4987	68.2
**Educational attainment of participant**		
Nil	1329	18.2
Primary	2187	29.9
Secondary/Higher	3797	51.9
**Educational attainment of Husband**		
Nil	1958	26.8
Primary	2119	29.0
Secondary/Higher	3236	44.2
**Employment**		
Yes	1066	14.6
No	6247	85.4
**Microcredit borrower**		
Yes	1881	25.7
No	5432	74.3
**Sex of household head**		
Male	6549	89.6
Female	764	10.4
**Household wealth status**		
Poor	2916	39.9
Average	1408	19.3
Rich	2989	40.9
**Has a say in own healthcare decision**		
Yes	834	11.4
No	6479	88.6
**Has a say in household spending**		
Yes	413	5.6
No	6900	94.4
**Aware of community clinic**		
Yes	5959	81.5
No	1354	18.5
**Received Antenatal care**		
Yes	4872	66.6
No	2441	33.4
**Has any family plan**		
Yes	3141	43.0
No	4172	57.0
**Heard family planning on radio**		
Yes	197	2.7
No	7116	97.3
**Heard family planning on TV**		
Yes	1889	25.8
No	5424	74.2
**Heard family planning in newspaper/magazine**		
Yes	263	3.6
No	7050	96.4

### Cross tabulation

The chi-squared test results presented in Table 2. show the factors associated with the utilization of institutional delivery. Less than one-third of the participants reported delivering at a health facility. Results showed that the rate of service utilization was higher among participants of urban origin, participants aged 15 to 29 years, participants with secondary/higher education qualifications, participants with husbands who have secondary/higher education qualifications, participants without microcredit membership, and participants living in wealthy households. Participants that were aware of available community clinic services, involved in family planning, had learned about family planning on the radio, and had received antenatal care services were also more likely to use institutional delivery services.

**Table 2:** Relative percentage of participants utilizing institutional delivery services across the explanatory variable groups, BDHS 2011.

Variables	Institutional delivery		*X*^2^	*p*-value
	No (%)	Yes (%)		
**Age**			5.292	0.021*
15–29	3919(70.6)	1630(29.4)		
30–49	1296(73.5)	468(26.5)		
**Residency**			512.229	<0.001*
Urban	1251(53.8)	1075(46.2)		
Rural	3964(79.5)	1023(20.5)		
**Educational attainment of participant**			671.280	<0.001*
Nil	1194(89.8)	135(10.2)		
Primary	1806(82.6)	381(17.4)		
Secondary/Higher	2215(71.3)	1582(41.7)		
**Educational attainment of Husband**			698.514	<0.001*
Nil	1711(87.4)	247(12.6)		
Primary	1694(79.9)	425(20.1)		
Secondary/Higher	1810(55.9)	1426(44.1)		
**Employed**			0.205	0.651
Yes	754(70.7)	312(29.3)		
No	4461(71.4)	1786(28.6)		
**Microcredit borrower**			50.07	0.001*
Yes	1461(77.7)	420(22.3)		
No	3754(69.1)	1678(30.9)		
**Sex of household head**			1.364	0.243
Male	4684(71.5)	1865(28.5)		
Female	531(69.5)	233(30.5)		
**Household wealth status**			970.936	<0.001*
Poor	2566(88.0)	350(12.0)		
Average	1095(77.8)	313(22.2)		
Rich	1554(52.0)	1435(48.0		
**Has a say in own healthcare decision**			1.638	0.201
Yes	579(69.4)	255(30.6)		
No	4636(71.6)	1843(28.4)		
**Has a say in household spending**			0.709	0.400
Yes	287(69.5)	126(30.5)		
No	4928(71.4)	1972(28.6)		
**Aware of community clinic**			25.217	<0.001*
Yes	1041(76.9)	313(23.1)		
No	4174(70.0)	1785(30.0)		
**Received ANC**			755.317	<0.001*
Yes	2973(61.0)	1899(39.0)		
No	2242(91.8)	199(8.2)		
**Has any family plan**			107.905	<0.001*
Yes	2041(65.0)	1100(35.0)		
No	3174(76.1)	998(23.9)		
**Heard FP on radio**			6.113	0.013*
Yes	125(63.5)	72(36.5)		
No	5090(71.5)	2026(28.5)		
**Heard FP on TV**			0.583	0.445
Yes	1360(72.0)	529(28.0)		
No	3855(71.1)	1569(28.9)		
**Heard FP in newspaper/magazine**			0.382	0.537
Yes	192(73.0)	71(27.0)		
No	5023(71.2)	2027(28.8)		

Notes: ANC = Antenatal care; FP = Family planning

* = Significant at p<0.05

The variables, which were found to be significant in chi-square tests, were retained for regression modeling to control for confounding effects.

After controlling for potential confounders, all variables except for microcredit membership and learning about family planning on radio were found be significant 3.of utilization of institutional delivery service. Table 3. illustrates that participants from rural areas had a 46.9% lower prevalence of institutional delivery compared to urban dwellwers (OR = 0.531; p<0.001; 95%CI:0.467–0.604), whilst participants between ages 30 and 49 years demonstrated a 23.6% higher utilization of institutional delivery compared to those aged 15–29 years (OR = 1.236; p = 0.006; 95%CI: 1.062–1.437). Moreover, participants with higher education were about twice as likely to deliver at a standard health facility compared to those without formal education (OR = 2.081; p<0.001; 95%CI: 1.650–2.624), and similarly, husbands with higher educational attainment demonstrated an approximately 71% higher utilization of institutional delivery in comparison to those without formal education (OR = 1.709; p<0.001; 95%CI: 1.412–2.069).

**Table 3:** Predictors of institutional delivery service utilization among Bangladeshi women, BDHS 2011.

Variables	*p*-value	OR	95%CI
**Age** (30–49) ^a^			
15–29	**0.014**	0.968	0.709–1.462
**Residency** (Rural) ^a^			
Urban	0.001*	1.842	1.616–2.099
**Educational attainment of participant** (Nil) ^a^^,048^			
Primary	0.048****	1.264	1.002–1.483
Secondary/Higher	<0.001*	2.065	1.649–2.639
**Educational attainment of husband** (Nil) ^a^			
Primary	**0.993**	1.392	0.958–2.412
Secondary/Higher	0.001*	2.048	1.321–1.937
**Household wealth status** (Poor) ^a^			
Average	0.004*	1.304	1.088–1.562
Rich	0.001*	2.476	2.109–2.906
**Aware of community clinic services** (No) ^a^			
Yes	0.014****	1.214	1.037–1.420
**Has any family plan** (Yes) ^a^			
**No**	0.001*	0.653	0.578–0.737
**Received Antenatal care** (Yes) ^a^			
**No**	<0.001*	0.261	0.221–0.308

Notes

^a^ = Reference category

* = Significant at <0.05.

Model adjusted for microcredit membership, sex of the household, having any say in healthcare decision making and household spending, receiving ANC, hearing about FP on TV. Employment status and hearing about FP in newspaper/magazine were not included in the model as p was greater than 0.25 in the bivariate test.

Wealth status was a significant predictor of institutional delivery service use (see Table 4.), with participants belonging to the highest economic stratum being more likely to receive skilled care compared to those in the lowest economic stratum (OR = 2.507; p<0.001; 95%CI: 2.118–2.968). In addition, households of average economic class were found to have a 27% higher utilization of institutional delivery compared to those of lower economic standing. (OR = 1.272; p = 0.011; 95%CI: 1.057–1.531), and participants who were aware of community clinical services had a prevalence of institutional delivery of about 18% (OR = 0.816; p = 0.012; 95%CI: 0.696–0.957) compared to those who were hardly aware of community clinic services. Lastly, participants who engaged in family planning were 1.553 times more likely to use institutional delivery services compared to those who did not use family planning, (OR = 1.553; p<0.001; 95%CI: 1.374–1.754) and participants that received antenatal care were 3.639 times more likely to utilize institutional delivery services than those who did not (OR = 3.639; p<0.001; 95%CI: 3.074–4.308)

**Table 4:** Predictors of institutional delivery service utilization among Bangladeshi women using conditional logistic regression.

Variables	*p*-value	OR	95%CI
**Age (15-29years)^a^**			
30-49years	0.006***	1.236	1.062–1.437
Residence (Urban)^a^			
Rural	<0.001***	0.531	0.467–0.604
Educational level (Nil)^a^			
Primary	0.055	1.256	0.995–1.586
Higher	<0.001***	2.081	1.650–2.624
Husband Educational level (Nil)^a^			
Primary	0.995	0.999	0.824–1.213
Higher	<0.001***	1.709	1.412–2.069
Household wealth (Poor)^a^			
Average	0.011***	1.272	1.057–1.531
Rich	<0.001***	2.507	2.118–2.968
Aware of community clinic services (No)^a^			
Yes	0.012***	0.816	0.696–0.957
Has any family planning (No)^a^			
Yes	<0.001***	1.553	1.374–1.754
Received antenatal care (No)^a^			
Yes	<0.001***	3.639	3.074–4.308
Microcredit borrower (No)^a^			
Yes	0.249	0.919	0.796–1.061
Heard family planning on radio (No)^a^			
Yes	0.082	1.352	0.962–1.901

^a^ = reference category

***significant at 0.05

Pseudo R^2^ = 0.209

## Discussion and conclusions

MMR and utilization of institutional delivery services are two of the most important metrics of weighing the progress towards the achievement of MDG5. Though Bangladesh has made remarkable strides in reducing MMR over the past two decades and is recognized as one of the nine countries on track to meeting MDG5 on time [21], it is lagging far behind in terms of institutional delivery service use and skilled birth attendance related indictors [22]. In comparison to a recent study based on data from the 2007 BDHS, our results indicate a twofold increase in institutional delivery prevalence, although in absolute terms, this is still low (from 14.7% in 2007 to 28.7% in 2011). This low institutional delivery service use in the country is indeed a surprising phenomenon considering the fact that MMR has declined at a faster pace in Bangladesh compared to its neighboring countries, such as India and Nepal, which have higher levels of institutional delivery utilization than Bangladesh [23].

This paradoxical situation has drawn attention from top-notch researchers like Sen and has been attributed to recent progress in economic and educational terms and the overall improvement in social transformation and national health indicators [24]. Our findings also corroborate this proposed link between socioeconomic progress and improved health outcomes. For example, our findings showed no correlation between sex of household head and participant’s choice of delivery services, which could imply that women from both male- and female-headed households are enjoying somewhat equal levels of freedom in maternal health decision-making. Results also revealed that the educational attainments of both women and their husbands are significant predictors of delivering at healthcare facilities, which is consistent with previous findings in India demonstrating that MMR was substantially higher among women who had no formal education [20]. Moreover, a study conducted in Matlab, Bangladesh in 2009 reported that the increased rate of female literacy was strongly associated with the sharp decline in maternal mortality in the region [25]. This association between education and health indicators is intuitively reasonable, as educated individuals tend to be more cautious of personal health issues, have higher self-efficacy, and exhibit better adherence to self-care and healthy behaviour [26]. Besides educational attainment, our study found that age (being below 30) and regional category (being of urban origin) were also positively associated with the choice of institutional delivery, which suggests the existence of demographic and regional patterns with respect to MHS utilization. These findings therefore highlight that investments into educational programs that take into account regional variations and region-specific needs could in the long-term be conducive to achieving maternal health-related goals in Bangladesh.

We also found a significant influence of household characteristics on choosing delivery location. Poverty is a strong predictor of population health status, and its impact is even more pronounced in the case of women’s general and reproductive health outcomes. A recent study based on the National Family Health Survey (NFHS-3) found that the use of antenatal care and safe delivery were notably lower among the socioeconomically disadvantaged states in India and concluded that household poverty was a significant barrier to MHS utilization [27]. These results could be explained by the fact that low-income households usually spend the bulk of their budget on food and consequently face difficult trade-offs regarding spending on education and healthcare. This proposition is supported by the association observed in our study between institutional delivery and the husband’s occupation type. Specifically, it was found that women whose husbands were engaged in blue- and white- collar professions had a higher likelihood of delivering at a health facility compared to those whose husbands were farmers. As occupation type is indicative of both education and income status, it represents a relevant predictor of economic accessibility to essential healthcare services. Therefore, national policymaking targeting income generation and poverty reduction, especially among the farming community in Bangladesh, is likely to have beneficial effects on improving MHS utilization and reducing maternal morbidity and mortality in the country.

As expected, participants who were aware of community clinics had an increased likelihood of delivering at health institutions. Besides the provision of basic nutrition and healthcare services, community clinics in Bangladesh routinely organize health education and family planning programs, which contribute to the adoption of healthy behaviours among communities, including adherence to immunization and supplementation programs, as well as use of contraception and family planning services. Family planning has been shown to be a key instrument for achieving all eight MDGs [28] and recommended as a priority element within the next global development agenda. Previous studies in Bangladesh have reported that the reduction in fertility alone has averted more than half of all maternal deaths between 2001 and 2010 [16], which suggests that scaling up family planning services through community clinics holds great promise for attaining MMR-related goals in the country. One study conducted in Ethiopia found that the odds of utilizing ANC and skilled delivery services were respectively 7 and 4.4 times higher among women who were aware of at least one danger sign of pregnancy [23,29]. Apart from family planning, community clinics also play an important role in raising awareness about pregnancy and obstetric complications. Furthermore, these clinics contribute to increasing MHS utilization, including ANC and institutional delivery, and are thus conducive to the prevention of maternal mortality [29,30], as women who attend antenatal services are generally more aware of the risks associated with childbirth and are as likely to deliver at healthcare institutions [27].

As our study was based on the 2011 DHS dataset, the study sample size was moderately high and the analysis included a wide range of variables. Limitations of the study however include the secondary nature of the data, the fact that its collection predates this analysis by several years, and the lack of control over the selection and measurement of the variables.

In conclusion, utilization of institutional delivery services is very low in Bangladesh (less than one in three women delivering at a health facility in 2011). As suggested by previous researchers [31,32,33,34], promoting the rate of institutional delivery should be regarded as a key strategy in efforts towards reducing the burden of maternal and infant mortality with the aim of attaining maternal and child health (MCH)-related goals in the country. This study concludes that focusing on female education, poverty reduction, and strengthening reproductive healthcare services through community clinics, with particular attention to rural communities, could substantially contribute to improving national maternal mortality prevention programs.

## Abbreviations

Antenatal care: ANC
Bangladesh demographic and health survey: BDHS
Health care professionals: HCPs
Maternal Healthcare Services: MHS
Millennium Development Goal: MDG
Postpartum hemorrhage: PPH
Low-and middle-income countries: LMICs
Traditional birth attends: TBAs
